# Strain-dependent grain boundary properties of n-type germanium layers

**DOI:** 10.1038/s41598-024-56282-0

**Published:** 2024-04-03

**Authors:** Kota Igura, Koki Nozawa, Takamitsu Ishiyama, Takashi Suemasu, Kaoru Toko

**Affiliations:** 1 Institute of Applied Physics, University of Tsukuba, 1-1-1 Tennodai, Tsukuba, Ibaraki 305–8573 Japan; 2 JSPS Research Fellow, 8 Ichiban-cho, Chiyoda-ku, Tokyo, 102–8472 Japan

**Keywords:** Electronic devices, Applied physics, Electrical and electronic engineering

## Abstract

Polycrystalline Ge thin films have attracted considerable attention as potential materials for use in various electronic and optical devices. We recently developed a low-temperature solid-phase crystallization technology for a doped Ge layer and achieved the highest electron mobility in a polycrystalline Ge thin film. In this study, we investigated the effects of strain on the crystalline and electrical properties of n-type polycrystalline Ge layers. By inserting a GeO_*x*_ interlayer directly under Ge and selecting substrates with different coefficients of thermal expansion, we modulated the strain in the polycrystalline Ge layer, ranging from approximately 0.6% (tensile) to − 0.8% (compressive). Compressive strain enlarged the grain size to 12 µm, but decreased the electron mobility. The temperature dependence of the electron mobility clarified that changes in the potential barrier height of the grain boundary caused this behavior. Furthermore, we revealed that the behavior of the grain boundary barrier height with respect to strain is opposite for the n- and p-types. This result strongly suggests that this phenomenon is due to the piezoelectric effect. These discoveries will provide guidelines for improving the performance of Ge devices and useful physical knowledge of various polycrystalline semiconductor thin films.

## Introduction

Although Ge is the oldest semiconductor used, it has once again attracted attention as its electrical and optical properties are useful for various next-generation electronics such as transistors^1–3^, solar cells^4,5^, optical communication^6–8^, and thermoelectric devices^9,10^. Particularly, there is an urgent need for a synthesis technology for Ge thin films on insulators for the following reasons: (i) although single-crystal (sc-) Ge is expensive, thinning the film can significantly reduce costs. (ii) Ge has a high optical absorption coefficient (~ 10^4^ cm^−1^ at 0.8 eV), so even a thin film can absorb sufficient light^11^. (iii) The leakage current in transistors due to the narrow bandgap can be solved by thinning the Ge film^3,12,13^. (iv) Owing to the low crystallization temperature and Young’s modulus, it can be synthesized on general-purpose substrates, such as glass and plastic^14–17^. Since most insulators are amorphous, Ge films synthesized directly on insulators become polycrystalline with various defects, including grain boundaries. Moreover, since the defects in Ge behave as acceptors^18–22^, all undoped polycrystalline (poly-) Ge films exhibit p-type conduction^23–27^. Furthermore, the activation rate of the n-type dopants in Ge is low^28–31^. This makes it difficult to control the Fermi level, which is important in all semiconductor devices.

In recent years, it has been discovered that in the solid-phase crystallization (SPC) of Ge thin films, increasing the density of amorphous Ge precursors or adding impurities significantly affects the crystallinity of the resulting poly-Ge thin films^32–34^. We achieved the lowest hole concentration (2 × 10^16^ cm^−3^) for a poly-Ge thin film^35^ and also realized control of n-type conduction by doping with impurities (Sb, As, and P) ^36^. The carrier mobility reached the highest value for a poly-Ge thin film (holes: 690 cm^2^ V^−1^ s^−1^, electrons: 450 cm^2^ V^−1^ s^−1^)^37,38^. The best performance of a low-temperature thin film transistor using a poly-Ge layer was also demonstrated^39,40^.

Based on these techniques, we have studied strains naturally applied to poly-Ge thin films^41^. The amount of strain mainly depended on the difference in the thermal expansion of the substrate. Although it was not sufficiently large to modulate the band structure of Ge^42^, it significantly affected the grain boundary barrier height (*E*_B_) of the p-type Ge thin film. In this study, we investigate the effects of strain on the crystallinity and electrical properties of n-type Ge thin films. The strain dependence of *E*_B_ in the n-type Ge thin films was inversely correlated with that in the p-type Ge thin films. This behavior suggests that the change in *E*_B_ owing to strain is due to the piezoelectric effect^43–46^.

## Experimental

Various substrates with different coefficients of thermal expansion (CTEs) were used to modulate widely the strain applied to the Ge layer, including SiO_2_, Si (111), CaF_2_ (001), and polyimide (PI). We denote the CTE difference between Ge and substrate as *Δα*; the values are presented in Table 1. Before thin-film deposition, the substrates were cleaned with acetone, methanol, and distilled water. We fabricated 15-nm thick GeO_*x*_ layers on the substrates using radio-frequency magnetron sputtering (base pressure: 4.0 × 10^–4^ Pa) at 10 sccm. Ar plasma (working pressure: 0.5 Pa) was used with the radio-frequency power set to 50 W. The GeO_*x*_ layer excluded the influence of the substrate-interface species and extracted the influence of the difference in CTEs on the Ge layer^37,41^. The samples were then air-transferred from the sputtering chamber to a molecular beam deposition system (base pressure: 5 × 10^−7^ Pa) within five minutes to avoid the reaction of GeO_*x*_ with air. Subsequently, the phosphorus-doped amorphous Ge layers were prepared using a Knudsen cell with a solid Ge source (purity: 99.999%). The Ge thickness was maintained at 200 nm, with a constant Ge deposition rate of 3.4 nm min^−1^. The phosphorus concentration in Ge was 2 × 10^20^ cm^−3^. The sample stage was heated at 125 °C during deposition to densify the amorphous Ge layer^33,38^. The samples were then loaded into a conventional tube furnace (Koyo Thermo Systems, KTF035N1) with N_2_ flow (purity: 99.9%, flow rate: 0.1 L min^−1^) and annealed at 400 °C for 50 h to induce SPC. The temperature was calibrated by placing a thermocouple directly on the tube furnace and was confirmed to be uniform within the sample stage.

**Table 1:** Coefficients of thermal expansion (CTEs) and CTE differences between Ge and substrate (*Δα*) at room temperature.

Substrate	CTE [10^–6^ K^–1^]	*Δα* [10^–6^ K^–1^]
SiO_2_	0.5	–5.3
Si	3.9	–1.9
CaF_2_	18.9	13.1
PI	27.0	21.2

Optical microscopy was employed during annealing using a Linkam 10042 D microscope with Keyence VH-5500. The resulting samples were evaluated using Raman spectroscopy, atomic force microscopy (AFM), scanning electron microscopy (SEM), electron backscatter diffraction (EBSD), and Hall-effect measurements. Raman spectra were measured using a JASCO NRS-5100 with a frequency-doubled Nd: YAG laser (wavelength: 532 nm, power: 0.5 mW, spot diameter: 5 μm), where the laser power was weak enough not to affect the crystal phase and peak shift. The penetration depth of the laser in poly-Ge was estimated to be approximately 30 nm from the absorption coefficient of Ge at the laser wavelength. The absolute Raman shift (detector resolution: 0.42 cm^−1^) was calibrated using the transverse optical phonon line (300 cm^−1^) of sc-Ge(100). The root mean square (RMS) values were measured by AFM using a Shimadzu SPM-9600 instrument. The SEM and EBSD were performed using a Hitachi High-Tech SU7000 instrument (voltage: 15 kV) equipped with an Oxford AZtec analysis attachment. Hall effect measurements using the Van der Pauw method were performed using an M91 FastHall and AX-2022041R with a 0.45 T permanent magnet.

## Results and discussion

**Figure 1:**
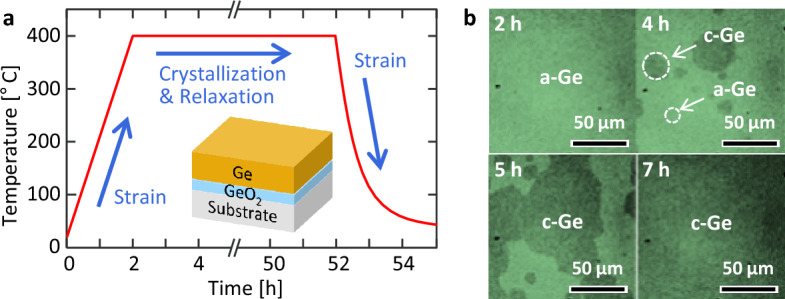
Annealing process of the samples. (**a**) Temperature profile and strain history in Ge. The inset shows the schematic of the sample structure. (**b**) Annealing-time evolution optical micrographs showing the crystal growth of the sample with a SiO_2_ substrate. The light- and dark-colored areas indicate amorphous (a-) and crystalline (c-) Ge, respectively.

Figure 1a shows how strain is introduced into the Ge layer during the annealing process. During temperature rise, strain is applied to Ge according to *Δα*. Subsequently, strain relaxation in Ge happens during the nucleation and grain growth at 400 °C as a dynamic process. During temperature reduction, strain is reintroduced into Ge according to *Δα*, which is in the opposite direction to that applied during the temperature rise. Figure 1b shows that the Ge crystallization progresses with increasing annealing time. According to the optical microscopy observations, crystallization was completed within 5 h at 400 °C for all samples. Therefore, the long-time annealing for 50 h would sufficiently relax the strain in Ge at 400 °C, leaving the strain only due to *Δα* in Ge after cooling.

**Figure 2:**
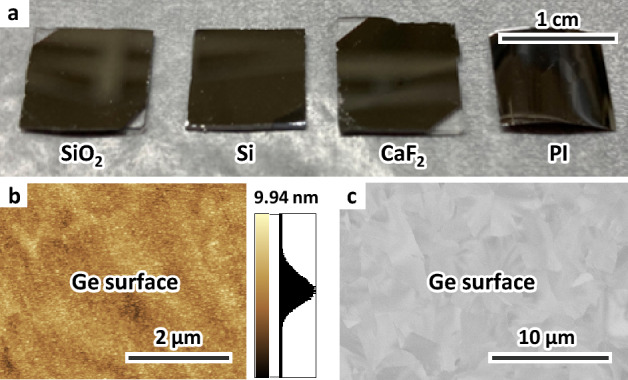
Sample appearance after annealing. (**a**) Photograph of the samples with an SiO_2_, Si, CaF_2_ and PI substrate. (**b**) AFM and (**c**) SEM images of the sample with a SiO_2_ substrate.

As shown in Fig. 2a, a uniform mirror-like Ge film was formed in all samples. The AFM image in Fig. 2b shows that the surface of the Ge layer is flat, which is one of the advantages of SPC. The SEM image in Fig. 2c shows a contrast on the order of micrometers. When considered together with the AFM results, the contrasts in the SEM image is due to the electron channeling effect: a phenomenon in which the penetration depth of an electron beam changes depending on the crystal orientation^47^. This suggests that the Ge layer has high crystallinity, which is similar in all samples.

**Figure 3:**
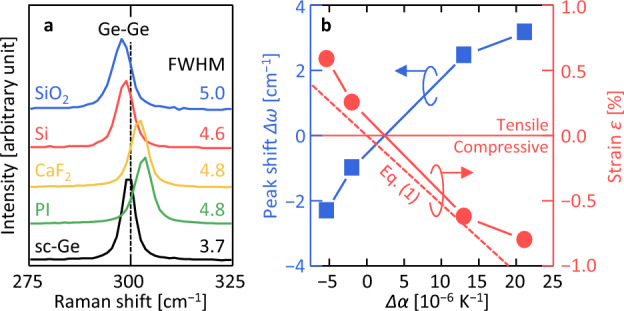
Raman spectroscopy analysis of the samples. (**a**) Raman spectra of the samples after annealing, where the crystal Ge peak position for a sc-Ge wafer is shown as a dotted line. The FWHM values of the Ge peaks are shown near each spectrum. (**b**) *Δω* and *ε* as functions of *Δα*, where *ε* > 0 corresponds to tensile strain and *ε* < 0 corresponds to compressive strain. The dotted line shows *ε*_th_ obtained from Eq. (1).

Figure 3a shows a sharp peak around 300 cm^−1^ caused by Ge crystals in the Raman spectra of all samples. Although the full width at half maximum (FWHM) of the Ge peaks was approximately the same, the Raman shift changed depending on the sample. Figure 3b shows the peak shift (*Δω*) from the Ge peak of the sc-Ge wafer in each sample. It can be seen that as *Δα* becomes larger, *Δω* becomes larger. The strain *ε* in the Ge layer is expressed as *ε* = *Δω* / *b* using the strain phonon coefficient *b*. The *ε* value of the Ge film on each substrate was calculated by substituting *b* =  − 395 cm^−1^, as proposed by Manganelli et al.^48^*.* A positive *ε* corresponds to tensile strain, and a negative *ε* corresponds to compressive strain. It can be seen that tensile strain is applied when *Δα* < 0, and compressive strain is applied when *Δα* > 0. Furthermore, the theoretical strain *ε*_th_ obtained from *Δα* is shown by a dotted line defined using

1 $$\varepsilon_{{{\text{th}}}} = \frac{\Delta \alpha \Delta T }{{1 {-} v}},$$

where *ΔT* is the difference between the annealing temperature (400 °C) and room temperature (300 K), and *ν* is the Poisson's ratio of the thin film^49^. The trend of *ε* roughly matches the trend of *ε*_th_, which indicates that the difference in CTEs with the substrate mainly causes the strain in the Ge layer. Moreover, *ε* showed a slightly higher value than *ε*_th_ in all samples. One possible reason for the difference could be that *Δα* was calculated as a constant, while CTE depended on temperature^50^. From the above, it can be seen that a reasonable strain is applied to the Ge film in response to *Δα*.

**Figure 4:**
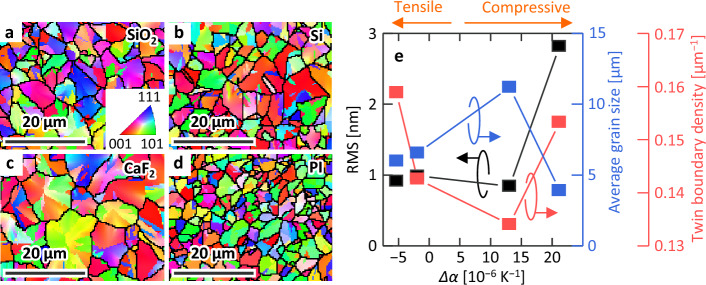
Crystal properties of the Ge layers. Inverse pole figure images from EBSD analyses for the samples with (**a**) SiO_2_, (**b**) Si, (**c**) CaF_2_, and (**d**) PI substrate. The color in crystal orientation maps indicates the crystal orientation (refer to legend in the inset). Grain boundaries are shown as black lines. (**e**) RMS, average grain size, and twin boundary density as a function of *Δα*.

Figures 4a–d show inverse pole figure images of the Ge layers obtained by EBSD. A random orientation was observed in all samples, and no difference in orientation was observed depending on the substrate. This is due to the insertion of an a-GeO_*x*_ layer at the Ge/substrate interface^37,41^. By contrast, the grain size depends on the type of substrate used. The RMS roughness of each sample was evaluated using AFM, as shown in Fig. 4e. Although the RMS roughness was similar for the samples with the SiO_2_, Si, and CaF_2_ substrates, it was higher for the sample with the PI substrate. Since these values are comparable to the RMS of each substrate, it can be seen that the Ge layer inherits the irregularities of the substrate. Figure 4e shows results summarizing changes in the average grain size and twin grain boundary density concerning *Δα*. As *Δα* increased, the grain size increased and then decreased, while the twin grain boundary density exhibited the opposite trend. It is known that both strain and roughness in thin films affect the nucleation frequency and growth rate^14,51–53^. The fact that the Ge grain size varied for the SiO_2_, Si, and CaF_2_ samples with similar roughness suggests that the strain applied to the a-Ge layer during temperature rise affects the crystal growth (Fig. 1a). Conversely, the small grains in the PI sample are explained as a pronounced interfacial inhomogeneous nucleation due to the rough substrate surface.

**Figure 5:**
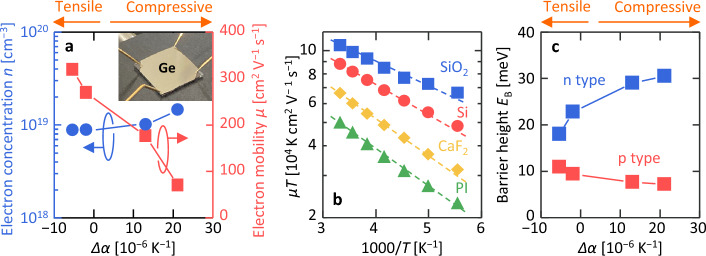
Electrical properties of the Ge layers. (**a**) *n* and *µ* as functions of *Δα*, where the inset shows the measurement setup. (**b**) Arrhenius plots of *μT* of the samples, where the dotted lines are the fitted lines used to derive *E*_B_. (**c**) *E*_B_ as a function of *Δα*. The data of p-type Ge layers were derived by recalculating the data in Ref. 41 with the Evans and Nelson model.

Figure 5a shows the results of the Hall measurements. Similar to the behavior of hole concentration in p-type Ge^41^, the electron concentration *n* was almost constant with respect to *Δα*. Conversely, as *Δα* increases, the electron mobility *µ* decreases. Generally, *µ* in polycrystalline semiconductors is limited by grain boundary scattering^54,55^, but this behavior is inconsistent with the grain size trend shown in Fig. 4. To clarify this, we calculated *E*_B_ based on the Evans and Nelson model^55^, where μ is defined as

2 $$\mu = \frac{Lq}{{k_{{\text{B}}} T}}\frac{{v_{r} }}{{1 + \frac{{v_{{\text{r}}} }}{{v_{{\text{d}}} }}}}{\text{exp}}\left( { - \frac{{E_{{\text{B}}} }}{{k_{{\text{B}}} T}}} \right),$$

where *L* is the grain size, *q* is the elementary charge, *v*_r_ is the recombination velocity, *v*_d_ is the drift–diffusion velocity, *k*_B_ is Boltzmann’s constant, and *T* is the measurement temperature. Therefore, we can obtain *E*_B_ from the slope of the Arrhenius plots against *µT*. Figure 5b shows the Arrhenius plots for *µT*. It can be seen that *µT* decreases monotonically as a function of 1000 / *T*, which means that *µ* is limited by grain boundary scattering. Figure 5c shows the *E*_B_ calculated from the slope of the regression line indicated by the dotted line in Fig. 5b. As *Δα* increased, *E*_B_ clearly increased. By contrast, the *E*_B_ of a similarly prepared undoped p-type Ge layer decreased with increasing *Δα*, as we have previously reported^41^. Thus, the poly-Ge layer strain significantly affects the electrical properties, especially *E*_B_.

**Figure 6:**
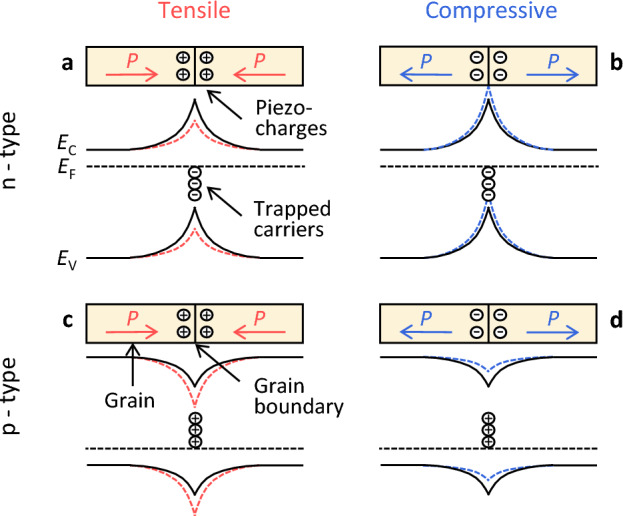
Strain effects on the grain boundary potential. Schematic of piezoelectric effects and band structures for the (**a**) tensile-strained n-type Ge, (**b**) compressive-strained n-type Ge, (**c**) tensile-strained p-type Ge, and (**d**) compressive-strained p-type Ge. The conduction (*E*_C_) and valence (*E*_V_) band curves are solid without strains and dashed with strains. *P* is the polarization vector generated by the strains in Ge.

The fact that *E*_B_ shows the opposite behavior with strain for the n-type and p-type layers strongly suggests that piezoelectric effects cause changes in *E*_B_^43–46^. Figure 6 illustrates the strain effects on the grain boundary barrier height of the Ge layer. In polycrystalline semiconductors, grain boundary defects act as carrier traps for electrons in n-type and holes in p-type, so that the Fermi level (*E*_F_) within the grain and at the grain boundary are equal. This phenomenon forms potential barriers that prevents carrier transport. When strain is applied to a poly-Ge layer, polarization vectors (*P*) are generated in the grains, corresponding to stain direction. When the charge induced near the grain boundary by the polarization (piezo-charge) and the trapped carriers at the grain boundary are of different types, *E*_B_ decreases due to charge compensation (Figs. 6a,d). In contrast, when the piezo-charge and trapped carriers are the same type, *E*_B_ increases (Figs. 6b,c). Thus, the relationship between the strain and *E*_B_ in the poly-Ge layer is consistent with the piezoelectric effect, although it is still a matter of speculation.

## Conclusions

We investigated the effects of the thermal strain caused by the substrate on the crystallinity and electrical properties of an n-type Ge layer. Using SiO_2_, Si, CaF_2_, and PI substrates, the amount of strain in the poly-Ge layer was modulated in the range from 0.6% to − 0.8%, where positive values correspond to tensile strain, and negative values correspond to compressive strain. Compressive strain expanded the grain size, reaching approximately 12 μm in the CaF_2_ substrate sample, while decreasing *μ*. We derived *E*_B_ from the temperature dependence of *μ* and clarified that a decrease in *μ* is due to an increase in *E*_B_. The behavior of *E*_B_ with strain was opposite between the n- and p-types, which strongly suggests that piezoelectric effect caused this phenomenon. These results will be crucial for controlling the properties of poly-Ge devices and various polycrystalline semiconductor thin films.

## Data Availability

The datasets used and/or analyzed in the current study are available from the corresponding author upon reasonable request.
